# Effect of the pH in the enrichment of X or Y sex chromosome-bearing sperm in bovine

**DOI:** 10.14202/vetworld.2019.1299-1303

**Published:** 2019-08-23

**Authors:** Nidhi P. Raval, Tejas M. Shah, Linz-Buoy George, Chaitanya G. Joshi

**Affiliations:** 1 Department of Animal Biotechnology, College of Veterinary Science and Animal Husbandry, Anand Agricultural University, Anand, Gujarat, India; 2 Department of Zoology, Biomedical Technology and Human Genetics, University School of Sciences, Gujarat University, Ahmedabad, Gujarat, India; 3 Gujarat Biotechnology Research Centre, Block B and D, 6^th^ Floor, MS Building, Sector – 11, Gandhinagar, Gujarat, India

**Keywords:** droplet digital polymerase chain reaction, spermatozoa, swim-up

## Abstract

**Background and Aim:**

Studies have shown that the pH of the vagina during the course of fertilization may influence the migration of X- and Y-bearing spermatozoa and thus leading to skewness in the sex of the offspring. Hence, this study was carried out to check the effect of the pH in the enrichment of X or Y sex chromosome-bearing sperm in bovine (*Bos indicus*).

**Materials and Methods:**

To check the effect of pH in the enrichment of X or Y sex chromosome-bearing sperm in bovine, we used buffers of various pH ranging from 5.5 to 9.0 for swim-up procedure of sperm sample and collected upper and bottom fraction from the same buffer and checked the abundance of X- and Y-bearing spermatozoa by droplet digital polymerase chain reaction using X- and Y-chromosome-specific DNA probe.

**Results:**

The abundance of X- and Y-bearing spermatozoa was not differed significantly in either of the fraction collected.

**Conclusion:**

Thus, it appears to be unlikely that an immediate impact of pH on sperm can be a solitary impact on the sex of offspring in bovine.

## Introduction

Various scientists have been researching if the natural framework deciding the sex of an infant is totally arbitrary or can be pre-selected. The idea of affecting the sex of one’s offspring has been of proceeding with intrigue for a long time. Researchers are still interested in exploring the fundamentals of inheritance of chromosome, maternal and paternal factors responsible for on sex determination of the offspring [1,2]. Despite the knowledge regarding differences between X and Y spermatozoa (for example, head measure [3], motile speed [4,5], DNA content [6,7], and pH susceptibility [8]) is available, still the definite physiological reasons for skewness in the proportion of spermatozoa carrying X or Y chromosomes with respect to environmental factors are not clear [9]. The only commercially available and effective method is using the difference in DNA content in the X- and Y-bearing spermatozoa [10,11]. Other methods where studies were carried out depending on the slight difference in density of X- and Y-bearing spermatozoa [12], Percoll and albumin gradient [13-15], modified swim-up method [16,17], using different Sephadex column [18], and presence of H-Y antigen [19,20]. Although contemporary strategies for physically separating X from Y-chromosome-bearing spermatozoa are presently very proficient, overall fertility rates following the use of sex-sorted sperm are not as noteworthy, despite numerous endeavors to improve them [21-24]. In the meantime, there are also studies from developmental science which gives the idea that the mammalian female could have a little impact on the sex of her posterity, and besides, that this impact could be pre-conceptual [25-27]. Provided above is true, this could go some way towards representing the putative inefficiencies when inseminated with sex-sorted sperm. Scientists believed that the X and Y sperm can be chosen based on different migration rates in acidic and basic media. Since the pH of human bodily fluid changes amid the menstrual cycle, scientists recommended that particular planning of intercourse in the female cycle could be utilized in choosing for either sex preceding conception [28]. Various studies, in general, show impact of pH on sperm motility, viability, and capacitation [29,30], but the alteration of pH can occur at both male and female level i.e. in semen sample as well as in vagina of female also, but its effect on skewness of sex ratio is still debatable field.

To get the offspring of our desire, people are interested in separating X- and Y-bearing sperm for further usage in artificial insemination and thereby meeting the need of humankind. Though with the continuous improvement [31,32] and advancement like use of optimized microfluidic system, better results in the human motile sperms before *in vitro* fertilization process [33] is achieved but still an alternative technique to enrich X and Y chromosome bearing sperm cells which would employ simpler and less expensive equipment is the need of the hour. Earlier study in mammals shows non-significant and contradictory results for impact of altering the pH on the migration of X- and Y-bearing sperm [34]. As the controversy persists regarding the effect of altering pH in skewness of sex ratio, this study will help by providing the evidence for whether it is possible to isolate X- and Y-bearing sperm by altering pH.

This study aimed to check whether pH can influence the sex proportion of posterity. To isolate the immediate impact of pH on the sperm from some other changes that may happen in the female’s conceptive tract at the season of ovulation, we incubated sperm in buffer of known pH to check whether it has any role in enrichment of either of the sex chromosome-bearing sperm.

## Materials and Methods

### Ethical approval

Semen utilized in this study was acquired from artificial vaginal ejaculate of Gir (*Bos indicus*) bulls as per Indian Animal Ethics Guidelines and with approval from the Institutional Animal Ethics Committee, Anand Agricultural University. Semen was collected from the Department of Veterinary Gynecology (CVASH, AAU, Anand) and transported to the research facility where it was washed with phosphate-buffered saline (PBS) (pH 7.4) to remove seminal fluid and other gelatinous materials.

### Semen sample processing

The first ejaculate from the two healthy Gir bulls was collected and the experiment was independently repeated 3 times. The semen samples were analyzed for various parameters, namely, concentration, motility, and viability before conducting the experiment. After taking the observations, the semen sample was gently mixed with PBS and centrifuged the tubes at 1600 rpm for 10 min. The procedure was repeated twice to wash the semen and finally resuspended the semen sample in small volume of PBS. After semen sample processing, 9×10^6^ sperm was taken for experimental setup. Conventional swim-up procedure was used to study the effect of pH on sperm survival and enrichment. Ringer’s lactate solution (Claris Otsuka, India) and Dulbecco’s Modified Eagle Medium (DMEM) – low glucose (Thermo Fisher Scientific, USA) medium were used for the pH experiment (pH 5.0-9.0). In any case, the addition of sperm samples slightly altered the pH of the original buffer solution so we prepared buffer such that the reference of the pH always indicates the pH of the final sperm sample buffer mixture.

Fifty milliliter conical centrifuge tubes were incubated at a 45° angle for 30-45 min for swim-up in a vertical rack in a 37°C CO_2_ incubator. After the incubation, aspirated the uppermost fraction and also the bottom fraction (BF) separately in 1.5 ml microcentrifuge tube for DNA extraction. DNA was extracted using phenol–chloroform method with little modification suggested in earlier studies [35].

### DNA extraction and quantification

Total DNA was quantified on Qubit 3.0 Fluorometer (Invitrogen, USA) using a DNA high sensitivity kit (Invitrogen, USA) and NanoDrop^®^ ND-1000 ultraviolet-visible spectrophotometer (Thermo Fisher Scientific, USA). The quality of the DNA was also checked on 0.8% agarose gel.

### Droplet digital polymerase chain reaction (ddPCR)

Kept ddPCR for both i.e. uppermost and bottom fractions of respective pH to check the ratio of X- and Y-bearing sperm in each collected fraction using DNA probe specific to X and Y chromosome. ddPCR assay was performed on Q×200 Droplet Digital PCR system (Bio-Rad, USA) using ddPCR Supermix for probes (no UTP). The assay was performed at absolute quantification mode and then abundance (copies/µl) was represented in percentage of X- and Y-chromosome-specific DNA probe F9 and sex-determining region Y (SRY), respectively. The ddPCR provides absolute quantification of the target DNA without the need to run standards which help in making this technique better for measuring target DNA copies. Additional advantage over a conventional quantitative PCR is high sensitivity and accuracy even with very low copy-number gene [36].

### Statistical analysis

Abundance for both F9 and SRY gene targets was measured using X- and Y-chromosome-specific DNA probe. The significant differences between the X- and Y-chromosome-bearing sperm population in the respective fractions were evaluated using Student’s t-test. A significance level of 0.05 was chosen. Data were presented as means ± standard error of the mean.

## Results

We checked the effect of pH of the media of various pH on the enrichment of either X- or Y-chromosome. When Ringer’s lactate solution was used for preparing buffer of different pH from 5 to 10, sperm was motile from pH 5.5, 6, 7, and 8 only, whereas when DMEM medium was used for preparing buffer of different pH from 5 to 10, sperm was motile from pH 5.5, 6, 7, 8, and 9 also.

Equal volumes of medium containing sperm were collected as upper and BF from each conical tube having medium of different pH after swim-up step. DNA was extracted from every fraction and after quantification and qualification check, an equal concentration of DNA was taken for further ddPCR.

**Figure-1:**
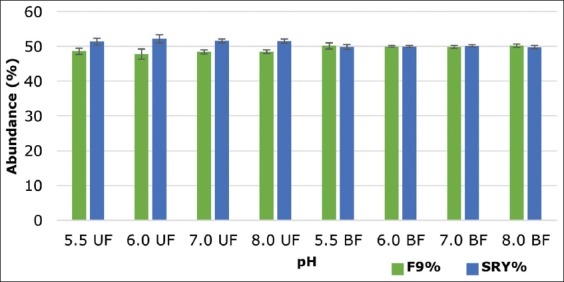
Abundance of X- and Y-chromosome-bearing sperm in upper and lower fractions collected from Ringer’s lactate solution having a different pH (UF: Uppermost fraction, BF: Bottom fraction). The data are displayed as mean ± standard error of the mean for three independent experiments.

**Figure-2:**
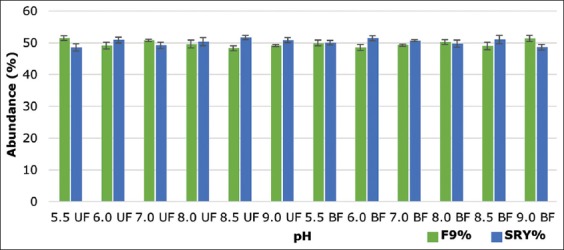
Abundance of X- and Y-chromosome-bearing sperm in upper and lower fractions collected from Dulbecco’s Modified Eagle Medium having a different pH (UF: Uppermost fraction, BF: Bottom fraction). The data are displayed as mean ± standard error of the mean for three independent experiments.

The ddPCR results representing the abundance of X- and Y-chromosome-bearing spermatozoa in the fractions collected from upper and bottom after swim-up showed statistically non-significant (p>0.05) enrichment of either X- or Y-chromosome-bearing spermatozoa on any of the fraction collected from medium, i.e. Ringer’s lactate solution (Figure-1) and DMEM medium (Figure-2) having different pH. The percentage difference between the abundance of X- and Y-bearing spermatozoa in the upper fraction collected was 2.76, 4.41, 3.18, and 3.05 whereas in BF 0.32, 0.01, 0.23, and 0.41 to pH 5.5, 6.0, 7.0, and 8.0, respectively, when the Ringer’s lactate solution was used. In case of DMEM medium, the percentage difference between the abundance of X- and Y-bearing spermatozoa in the upper fraction collected was 2.89, 1.76, 1.55, 0.79, 3.37, and 1.70 whereas in BF 0.09, 2.87, 1.33, 0.49, 2.02, and 2.76 to pH 5.5, 6.0, 7.0, 8.0, and 9.0, respectively. This would suggest that X and Y sperm are not differentially influenced by just the pH in the female cervix at ovulation.

## Discussion

It has been observed that at the time of fertilization, the pH of the vagina may play a role in the selection of X- or Y-bearing sperm and thereby affects the sex of the offspring [37]. The pH of the vagina is moderately acidic which varies from 3.8 to 4.5 [38]. Thus, studies have been done and patented, wherein sperm cells were put in an environment having physiological pH and at the same time contacting the sperm cells with at least one extra subenvironment with differing pH to enrich the population of specific type of sperm cells [39,40].

In recent transcriptome study wherein single female pig, using the bilateral laparoscopic insemination, the X- or Y-sperm populations were introduced into the two separate oviducts and they observed specific transcriptomic responses w.r.t X- and Y-chromosome-bearing sperm cells. Hence, they suggested the role of oviduct as biological sensor which can identify the sperm and then provide the respective modified environment hinting its role in gender biasing mechanism or maternal influence on gender [41].

In human, researcher has observed that the Y-chromosome-bearing spermatozoa were more vulnerable to fluctuations in physiological and sperm storage conditions than X-chromosome-bearing spermatozoa [42]. Hence, we have used different pH to check the enrichment of either X- or Y-chromosome-bearing spermatozoa in the respective fraction. However, we could not find any significant skewness in X- and Y-chromosome-bearing sperm population.

Studies on human showed the positive results for enrichment of X bearing spermatozoa upon incubation in acidic medium, increased temperature and raised reactive oxygen species (ROS) levels leading to suggesting the potential for semen sexing by exploring the different physiological parameters [8].

Studies have shown in boar spermatozoa that at high external pH, internal pH also increased and motility of epididymal spermatozoa was initiated, whereas motility of ejaculated spermatozoa was less dependent on external pH and altered very slightly by the changes in the internal pH [43]. Spermatozoa swim in different physiochemical conditions and are exposed to changes in temperature, pH, and ROS levels for as long as 1 week preceding fertilization [44].

Previously, studies have been done with human spermatozoa on checking the difference in the migration of X- and Y-bearing spermatozoa using capillary tubes which had a pH adjusted Tyrode’s solution. They used specific stains which stain the long arm of the Y chromosome to determine the chromosome present in the migrated sperm cells. In this study also, there is no enrichment of either X or Y chromosome bearing spermatozoa regardless of difference in the pH media used [45]. Whereas, studies carried out on rabbits exhibited a changed sex proportion in the posterity of rabbits in connection to the pH of the vagina at the season of mating. At the point, when the cervical pH was 6.5-7.3, there was a transcendence of females; there was no distinction in sex proportion with a pH of 7.3-7.5, and at pH 7.5-8.3, there was an abundance of males [37].

Another study in human and rabbit spermatozoa used cation and anion exchange resins of different ionic strength and examined for the enrichment of the specific sperm cells by ion-exchange column chromatography. Apparently, by this treatment, only dead spermatozoa of rabbit got filtered out [46].

All these methods depend on sperm arbitrarily interacting with the favored environment, but some of the environments provided may be negative to sperm motility and viability [47,48].

## Conclusion

The percentage of X- or Y-bearing sperm migrating during swim-up procedure was not influenced by the pH of the media in bovine sperm samples. Additional studies are, therefore, necessary to examine whether external pH is involved in the enrichment of X- and Y-bearing sperm by altering pH along with various other physiochemical factors in bovine.

## Authors’ Contributions

NPR: Conducted the experiment, acquisition of data, and drafting of the manuscript. TMS: Supervised the experiment, revised the manuscript, and advised on this study. LBG: Advised in the design of the experiment, data analysis, and interpretation. CGJ: Research project investigator, research and ethical clearance preparation, designed the study, analyzed statistical data, proofread the manuscript. All the authors have read and approved the final version of the manuscript.
